# The occurrence of non-melanoma malignant skin lesions and non-cutaneous squamous-cell carcinoma among metastatic melanoma patients: an observational cohort study in Denmark

**DOI:** 10.1186/s12885-016-2315-0

**Published:** 2016-05-03

**Authors:** Haojie Li, Lars Pedersen, Mette Nørgaard, Sinna P. Ulrichsen, Sandra K. Thygesen, Jeanenne J. Nelson

**Affiliations:** Worldwide Epidemiology, R&D, GlaxoSmithKline, 1250 South Collegeville Rd, Collegeville, PA 19426 USA; Department of Clinical Epidemiology, Aarhus University Hospital, Olof Palmes Allé 43 – 45, Aarhus N, DK-8200 Denmark; Worldwide Epidemiology, R&D, GlaxoSmithKline, 5 Moore Drive, Research Triangle Park, NC 27709-3398 USA

**Keywords:** Incidence, Cutaneous melanoma, Basal-cell carcinoma (BCC), Cutaneous squamous-cell carcinoma (cuSCC), Bowen’s disease, keratoacanthoma (KA), Actinic keratosis (AK), Non-cutaneous squamous-cell carcinoma (non-cuSCC), Population-based, Danish Cancer Registry, National Pathology Registry

## Abstract

**Background:**

Inhibitors of mutant BRAF are emerging as standard of care in patients with metastatic melanoma who carry relevant oncogenic mutations. However, BRAF inhibitors are found to induce cutaneous squamous cell carcinoma (cuSCC). Population-based background rates of cuSCC and non-cutaneous squamous cell carcinoma (non-cuSCC) in the metastatic melanoma population may contextualize safety signals from randomized clinical trials or the clinics. However, these background rates are lacking.

**Methods:**

We conducted a historical cohort study to evaluate the background rates of new-onset non-melanoma skin lesions and non-cuSCC among 2,814 metastatic malignant melanoma patients diagnosed in 1997–2010, identified through the Danish Cancer Registry and the National Pathology Registry. Patients were excluded if they had a history of cancer before the metastatic melanoma diagnosis, other than skin cancers. We determined the incidence of non-melanoma malignant skin lesions and non-cuSCC that occurred post metastatic melanoma diagnosis, censoring patients at death, emigration, or December 31, 2011 (end of study period), whichever came first.

**Results:**

The median age at metastatic melanoma diagnosis was 64 years. Over 40 % of patients died within one year of metastatic diagnosis and ~70 % died within 5 years. The percentages of patients with prior history or prevalent disease at metastatic melanoma diagnosis included: 8.6 % with cuSCC or basal cell carcinoma (BCC), 3.9 % with actinic keratosis (AK), and 0.7 % with Bowen’s disease. No patients had past or current non-cuSCC per study exclusion criterion. The incidence of non-melanoma skin lesions during the 6 months post-metastatic melanoma diagnosis was as follows: BCC, 1.8 % (42.5 per 1000 person-years [PY]); AK, 0.8 % (18.6 per 1000 PY); cuSCC, 0.1 % (1.7 per 1000 PY); Bowen’s disease, 0.04 % (0.8 per 1000 PY); and keratoacanthoma (KA), 0 %. Non-cuSCC was observed in 3 patients (0.1 %; 2.5 per 1000 PY) at 3 sites: bronchi, heart and lung.

**Conclusion:**

CuSCC and non-cuSCC were rare events among metastatic melanoma patients.

## Background

The incidence of cutaneous melanoma has been rising during the last four decades in white populations worldwide [1]. Although it is highly curable with surgery if detected in its earliest stages, prognosis for metastatic cutaneous melanoma patients has been historically poor, with a median overall survival in the range of 6–10 months. This poor survival was due to limited treatment options and efficacy - mainly alkylating agents, dacarbazine and temozolomide, and immunotherapy with IL-2 and/or interferon-alpha (IFN-α) [2]. Recently, treatment with immunotherapy or inhibition of the mitogen-activated protein kinase (MAPK) pathway has demonstrated clinical benefit by prolonging OS and progression-free survival (PFS) in randomized trials [3–7].

However, non-melanoma skin cancers — well-differentiated cutaneous squamous-cell carcinomas (cuSCC) and keratoacanthomas (KA) — have developed in approximately 11 % to 30 % of patients treated with type I BRAF inhibitors such as dabrafenib and vemurafenib [3, 8–12]. The incidence of cuSCC in vemurafenib-treated patients was 24 % and mostly occurred early in the course of treatment, with a median time to the first appearance of 7 to 8 weeks [8]. Approximately one third of the patients who experienced cuSCC had more than 1 event, with a median time of 6 weeks between occurrences. Potential risk factors associated with cuSCC for those vemurafenib-treated patients have been shown to be older age (≥65 years), prior skin cancer, and chronic sun exposure. A prospective observational study of patients enrolled in the phase I ⁄II clinical trials of dabrafenib revealed 18 cuSCCs occurred in eight out of 41 patients (20 %) [10]. Patients who developed cuSCCs were substantially older than the rest of the study population (median age 62 vs. 40 years). Most cuSCCs (77 %) appeared between weeks 6 and 24 following commencement of therapy on both sun-damaged and non-sun-damaged skin.

It is well established that actinic keratoses (AKs) are premalignant lesions that can progress to cuSCCs at a rate of approximately 10 % per year [13, 14]. Similar to AKs, KAs may also belong to a spectrum of premalignant lesions that can transform into cuSCC over time [15]. The histologic similarities of KAs and cuSCCs, including infiltration and cytologic atypia [13, 16–19], and the reports of KAs metastasizing also support the idea that KAs are a type of well differentiated cuSCC [17, 20].

The background rates of incident malignant skin lesions among melanoma patients are lacking in the literature. Such data will help to contextualize emergent safety signals observed in clinical trials or the clinics among metastatic melanoma patients. We therefore examined the incidence rates of non-melanoma malignant skin lesions and non-cuSCC in a population-based study of metastatic melanoma patients in Denmark.

## Methods

### Study design

This was a historical observational cohort study among patients diagnosed with metastatic (stage IV) melanoma, based on prospectively collected data obtained from population-based medical databases in Denmark. According to Danish legislation, purely registry-based studies do not need permission from an ethical board. Our study was approved by the Danish Data Protection Agency (Jr no 2009-41-3653). Moreover, we were granted permission to abstract information from the medical files without obtaining informed consent by the National Board of Health.

The Danish National Health Service provides tax-supported health care for all inhabitants of Denmark. The Danish Cancer Registry (DCR) established in 1943 contains records of all incident cancers diagnosed in living patients or identified through autopsy. The 10th revision of the International Classification of Diseases (ICD-10) has been used to code tumors since 1978. DCR files include information on cancer type, site, morphology, and cancer history. It has been found to have a high degree of completeness [21, 22] and the proportion of morphologically verified tumors is 89 % [23]. The National Pathology Registry (NPR), which has been nationwide since 1997, contains data on type of pathological specimens, procedures, pathological tests and results, and the diagnoses assigned. Diagnoses are coded according to the SNOMED classification. The Danish Civil Registration System (CRS) has recorded data on residency, vital status, and marital status for the entire Danish population since 1968 [24]. The data on death (yes/no) are more than 99 % complete and very accurate [25].

### Study population

We identified all patients (age ≥18 years) diagnosed with metastatic (stage IV) melanoma from 1997 to 2010 in Denmark. Eligible patients included those who had initial diagnoses of metastatic (stage IV) cutaneous melanoma during 1997 to 2010 and those who had earlier stage melanoma at initial diagnosis who then progressed to metastatic (stage IV) melanoma during 1997 to 2010. Only those who had melanoma as their first primary cancer were included. Patients were excluded if they had a history of cancer, other than melanoma, basel-cell carcinoma (BCC), cuSCC, Bowen’s disease, AK and KA, before the metastatic melanoma diagnosis.

Metastatic melanoma patients were identified using two national databases ― the DCR and the NPR, based on the ICD-10 Diagnosis Code and SNOMED Morphology Codes (Appendix). The DCR documents the initial diagnoses of all primary cancers; the NPR captures a large number of metastatic melanomas identified during the follow-up post initial melanoma diagnosis, although it is not 100 % complete. The index date for a patient was defined as the date of metastatic melanoma diagnosis. The Danish Civil Registration Number, a unique identification number assigned to every Danish citizen at birth or immigration, was used to link the DCR and NPR to the CRS for obtaining data on patient demographics, medical history, immigration, and death. All metastatic melanoma patients were followed until death, emigration, or end of study period, i.e., December 31, 2011.

### Outcome definitions and measures

Overall survival status for these patients was described at 3, 6, 12, 36, and 60 months after metastatic melanoma diagnosis. Non-melanoma malignant skin lesions and non-cutaneous squamous-cell carcinoma were identified over the continuous intervals of 1, 2, 3, 6, 9, and 12 months after the index date, and over the total follow-up. Events of cuSCC, BCC, Bowen’s disease, AK, and KA were identified with SNOMED codes (Appendix), with restriction to the non skin topology site.

### Validation of metastatic melanoma diagnosis in the NPR

Medical charts were reviewed for a random sample of 65 metastatic melanoma patients, with a registration of metastatic melanoma (including lymph node metastases) in the NPR. Of these, 40 (62 %) patients were admitted to Department of Oncology in Aarhus and 25 (38 %) patients were admitted to the Department of Plastic Surgery in Aalborg. Medical records for one patient from Aalborg could not be located. Of the remaining 64 patients, all had metastatic cutaneous melanoma according to the medical records, yielding a positive predictive value (PPV) of 100 % (95 % confidence interval [CI]: 96.2-100.0).

### Statistical analysis

Mean, standard deviation, median, and range were derived for continuous variables, and number and proportion were described for categorical variables. All patients were followed from date of metastatic melanoma diagnosis until death, migration out of Denmark, or the end of study period (i.e., December 31, 2011). Kaplan-Meier survival curves were derived to describe time to death over the 3, 6, 12, 36, and 60 months after metastatic melanoma diagnosis. Patients were considered as censored if they were lost during the follow-up or if there was no enough follow-up time allowed at the end of the study period. Mortality rate and the CIs were calculated using the method outlined by Simon et al. [26].

We described the number and proportion (and 95 % CI) of patients with a history or pre-existing non-melanoma malignant skin lesions (i.e., BCC, cuSCC, Bowen’s disease, AK and KA, eligible) prior to metastatic melanoma diagnosis. None of these melanoma patients had a history or pre-existing non-cuSCC, by study design.

To be consistent with most clinical trial studies, for the current study, the incidence analysis on non-melanoma malignant skin lesions was first conducted among patients regardless of whether they had a history or pre-existing non-melanoma malignant skin lesions. The incidence analysis was further conducted among patients who had no history or pre-existing non-melanoma malignant skin lesions. The incidence analysis of non-cuSCC was conducted among all eligible patients. The sites of non-cuSCC were described. We described cumulative incidence in proportion, and incidence rate in person-year of non-melanoma malignant skin lesions (or non-cuSCC) over the continuous intervals of 0–1, 0–2, 0–3, 0–6, 0–9, 0–12 months (i.e., 0–30, 0–60, 0–90, 0–180, 0–270, 0–365 days), and over the total follow-up, following metastatic melanoma diagnosis. The numerator was the number of metastatic melanoma patients with non-melanoma malignant skin lesions (or non-cuSCC) occurring during the specified time period post metastatic melanoma diagnosis. For the determination of cumulative incidence, the denominator was the number of eligible metastatic melanoma patients. For the determination of incidence rate, the denominator was the total person-years of eligible metastatic melanoma patients, with the person-year for each patient calculated as the time from metastatic melanoma diagnosis to date of the event, death, last contact, or end of the study period, whichever occurred first. The incidence analyses were further stratified by age at diagnosis (18- < 65, 65- < 75, 75- < 85, and 85+ years).

All analyses were further stratified and compared between two subgroups, patients with metastatic disease at initial diagnosis and patients diagnosed with early stage melanoma initially who then progressed to metastatic stage during the follow-up. All analyses were done using SAS 9.2 software (Cary, NC, USA).

## Results

During 1997–2010, 3,669 metastatic (stage IV) melanoma patients were identified from the DCR and NPR, including 855 (23 %) patients with a history of cancer, other than cutaneous melanoma, BCC, cuSCC, Bowen’s disease, AK, and KA, before the metastatic melanoma diagnosis (Table 1). Compared to patients with a history of cancer, those without a history of cancer (*n* = 2,814) were younger (median age = 64 vs. 69 y) at metastatic melanoma diagnosis. A greater proportion of patients with a history of cancer died (87.6 %) with a median time to death of 5.7 months, compared to those without a history of cancer (74.4 %, median time to death = 9.7 months).

**Table 1:** Patient characteristics: comparison between those with (excluded) and without (included) prior history of cancer^a^

	With a history of cancer		Without a history of cancer	
	N	%	N	%
Total N	855	100.0	2814	100.0
Age (y) at diagnosis^b^				
Mean (SD)	67.9 (12.9)		63.3 (15.4)	
Median (range)	69 (27–96)		64 (19–103)	
Male	425	49.7	1540	54.7
Born in Denmark	836	97.8	2727	96.9
Site of metastasis^b^				
Skin	289	33.8	1527	54.3
Soft tissue	36	4.2	166	5.9
Liver	215	25.1	115	4.1
Lung	35	4.1	89	3.2
GI Tract	38	4.4	64	2.3
Brain	35	4.1	59	2.1
Bone	16	1.9	39	1.4
Salivary gland	12	1.4	34	1.2
Mamma	10	1.2	28	1.0
Cytology, pleura	4	0.5	17	0.6
Mucous membrane	6	0.7	6	0.2
Gallbladder/Pancreas	2	0.2	5	0.2
Vulva	4	0.5	5	0.2
Unspecified or unknown	153	17.9	660	23.5
Death^b^	749	87.6	2093	74.4
Time (mo) to death^b^, Median (range)	5.7 (0–156)		9.7 (0–176)	

^**a**^Patients were excluded if they had a history of cancer, other than melanoma, basal cell carcinoma, cutaneous squamous cell carcinoma, Bowen’s disease, actinic keratoses, and keratoacanthoma, before the metastatic melanoma diagnosis

^b^ *P*-value < 0.0001 for age at diagnosis (T-test), time to death (Wilcoxon rank sum test), number of death and site of metastasis (Chi-squared test)

Of 2,814 metastatic melanoma patients who had no history of cancer (i.e., other than cutaneous melanoma, BCC, cuSCC, Bowen’s disease, AK and KA), 1,313 (47 %) patients were initially diagnosed with stage IV melanoma, and 1,501 (53 %) patients were initially diagnosed with earlier stage melanoma and then progressed to stage IV at a later date (Table 2). Over time from 1997 to 2010, the proportion of patients with stage IV melanoma as the initial diagnosis decreased from 64.8 % to 40.6 %. Of these, 2,093 (74 %) patients died during the observation period (by December 31, 2011) and the overall mortality rate was 289.2 (95 % CI: 277.1-301.9) per 1000 person-years (PY). Among those who died, the median duration from metastatic disease diagnosis to death was 8.6 months for those diagnosed initially with stage IV melanoma and 10.6 months for those who progressed to stage IV at a later date.

**Table 2:** Characteristics for eligible metastatic melanoma patients who had no history of cancer^a^

	Overall		Stage IV at initial diagnosis		Progressed to stage IV	
	N	%	N	%	N	%
Total N	2814	100.0	1313	100.0	1501	100.0
Year at diagnosis^b^						
1997-2000	596	21.2	386	64.8	210	35.2
2001-2005	1015	36.1	438	43.2	577	56.8
2006-2010	1203	42.8	489	40.6	714	59.4
Age (y) at diagnosis						
Mean (SD)	62.8 (15.4)		63.2 (14.9)		62.4 (15.9)	
Median (range)	64 (19–103)		64 (21–97)		63 (19–103)	
18- < 40 yrs	240	8.5	101	7.7	139	9.3
40- < 55 yrs	561	19.9	249	19.0	312	20.8
55- < 65 yrs	669	23.8	327	24.9	342	22.8
65- < 75 yrs	629	22.4	292	22.2	337	22.5
75- < 85 yrs	536	19.0	270	20.6	266	17.7
85+ yrs	179	6.4	74	5.6	105	7.0
Male^b^	1540	54.7	679	51.7	861	57.4
Born in Denmark	2727	96.9	1269	96.6	1458	97.1
Site of metastasis^b^						
Skin	1527	54.3	515	39.2	1012	67.4
Soft tissue	166	5.9	69	5.3	97	6.5
Liver	115	4.1	47	3.6	68	4.5
Lung	89	3.2	37	2.8	52	3.5
GI Tract	64	2.3	33	2.5	31	2.1
Brain	59	2.1	21	1.6	38	2.5
Bone	39	1.4	19	1.4	20	1.3
Salivary gland	34	1.2	10	0.8	24	1.6
Mamma	28	1.0	15	1.1	13	0.9
Cytology, pleura	17	0.6	8	0.6	9	0.6
Gallbladder/Pancreas	5	0.2	3	0.2	2	0.1
Vulva	5	0.2	3	0.2	2	0.1
Mucous membrane	6	0.2	4	0.3	2	0.1
Unspecified or unknown	660	23.5	529	40.3	131	8.7
Death^b^	2093	74.4	1003	76.4	1090	72.6
Time (mo) to death^b^, median (range)	9.7 (0–176)		8.6 (0–176)		10.6 (0–160)	
Mortality rates (95 % CI), per 1000 person-years	289.2 (277.1 – 301.9)		303.2 (285.0 – 322.5)		277.5 (261.5 – 294.4)	

^a^Patients were excluded if they had a history of cancer, other than melanoma, basal cell carcinoma, cutaneous squamous cell carcinoma, Bowen’s disease, actinic keratosis and keratoacanthoma, before the metastatic melanoma diagnosis

^b^Chi squared test P value: year at diagnosis (<0.0001), gender (0.003), site of metastasis (<0.0001) and number of death (0.02); Wilcoxon rank sum test *P*-value: time to death (<0.05)

**Fig. 1:**
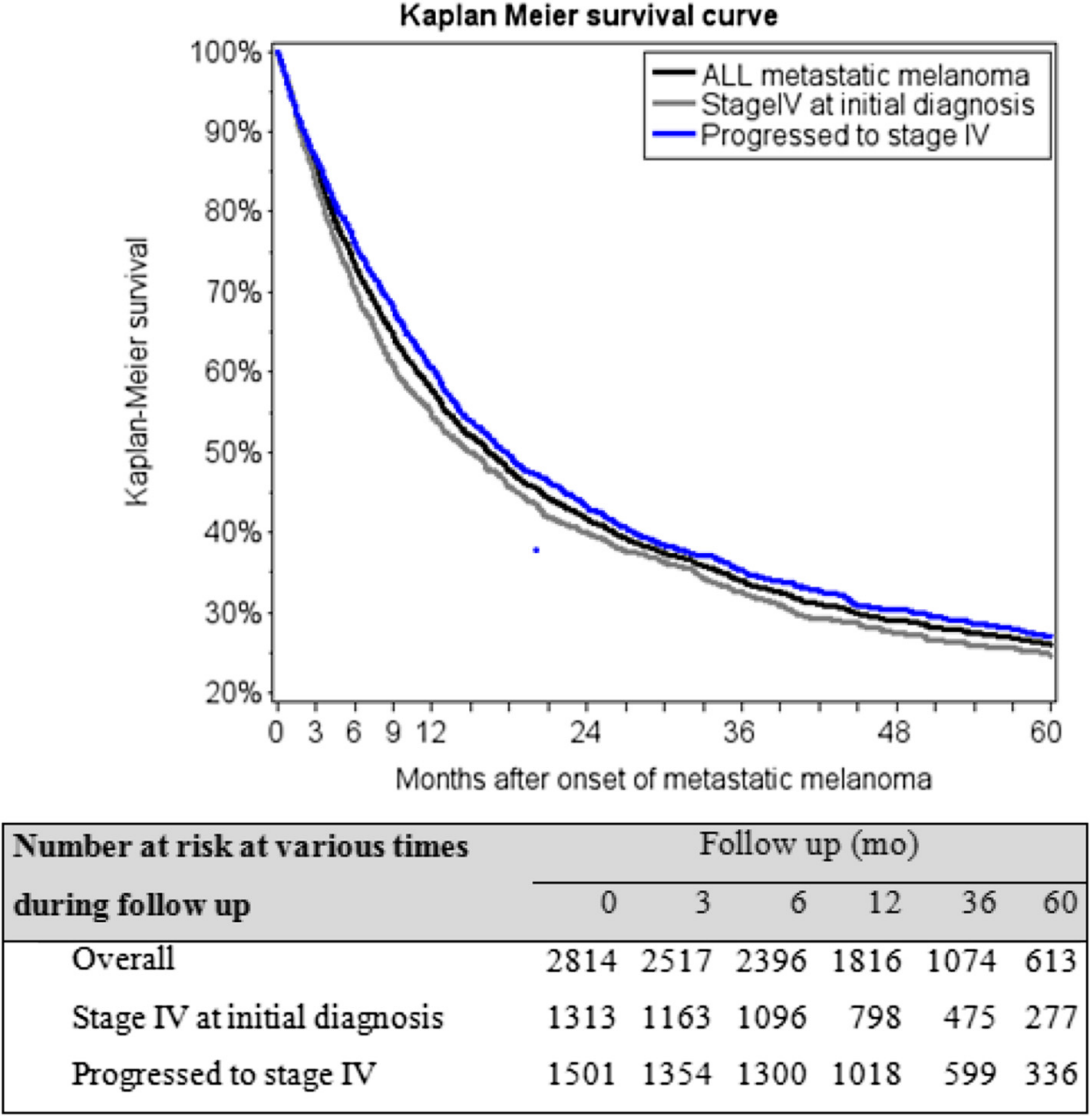
Kaplan Meier overall survival curve for metastatic melanoma patients post metastatic disease diagnoses. Figure Legend: Figure 1 shows the overall survival (in months) after onset of metastatic melanoma, presented for all patients, patients with Stage IV melanoma at initial diagnosis, and patients who had initial earlier stage melanoma then progressed to stage IV melanoma. The numbers of patients at risk at 0, 3, 6, 12, 36, and 60 months for each of these three groups are shown in the table immediately below the survival curves

The Kaplan-Meier curve (Fig. 1) illustrates the overall survival for patients with metastatic cutaneous melanoma. The overall 1-year survival was 58 % (95 % CI: 56 %-59 %) among 2,814 patients. The 1-year survival was slightly lower among patients who were diagnosed with stage IV melanoma initially (54 %, 95 % CI: 52 %-57 %), compared to those who had initially early stage melanoma and progressed to stage IV at a later date (60 %, 95 % CI: 58 %-63 %). The 5-year survival was 26 % (95 % CI: 24 %-28 %) overall, 25 % (95 % CI: 22 %-27 %) for patients who were diagnosed with stage IV melanoma initially, and 27 % (95 % CI: 25 %-30 %) for those who had initially early stage melanoma and progressed to stage IV at a later date.

Of the 2,814 metastatic melanoma patients with no prior history of other cancers, 8.6 % (95 % CI: 7.6 %-9.7 %) had a history or pre-existing cuSCC or BCC, 3.9 % (95 % CI: 3.2 %-4.6 %) had a history or pre-existing AK, and less than 1 % of these patients had a history or pre-existing Bowen’s disease or KA at metastatic melanoma diagnosis (Table 3). Patients who had initial early stage melanoma who progressed to stage IV disease at a later date had a higher prevalence of SCC/BCC (10.7 %; 95 % CI: 9.2 %-12.4 %) and AK (4.9 %; 95 % CI: 3.9 %-6.0 %) than those who had stage IV metastatic melanoma at initial diagnosis (SCC/BCC, 6.2 %, 95 % CI: 5.0 %-7.7 %; and AK, 2.7 %, 95 % CI: 2.0 %-3.7 %).

**Table 3:** History or pre-existing non-melanoma malignant skin lesions prior to metastatic melanoma diagnosis^a^

Outcome	Overall (*N* = 2814)		Stage IV at initial diagnosis (*N* = 1313)		Progressed to stage IV (*N* = 1501)	
	n	% (95 % CI)	n	% (95 % CI)	n	% (95 % CI)
cuSCC or BCC	243	8.6 (7.6-9.7)	82	6.2 (5.0-7.7)	161	10.7 (9.2-12.4)
Bowen’s disease	21	0.7 (0.5-1.1)	11	0.8 (0.4-1.4)	10	0.7 (0.3-1.2)
KA	19	0.7 (0.4-1.0)	9	0.7 (0.3-1.2)	10	0.7 (0.3-1.2)
AK	109	3.9 (3.2-4.6)	36	2.7 (2.0-3.7)	73	4.9 (3.9-6.0)

^a^Abbreviation: BCC, basal cell carcinoma; cuSCC, cutaneous squamous cell carcinoma; AK, actinic keratosis; and KA, keratoacanthoma

The cumulative incidence and incidence rates (Table 4) of non-melanoma malignant skin lesions are presented for all metastatic melanoma patients, including those who had a history of or pre-existing non-melanoma malignant skin lesions. Four patients died on the index date, thus leaving 2,810 patients for the incidence analysis. During the first 6 months post diagnosis, the cumulative incidence (and incidence rate) for all metastatic melanoma patients was 0.1 % (1.7 per 1000 PY) for cuSCC, 1.8 % (42.5 per 1000 PY) for BCC, 0.04 % (0.8 per 1000 PY) for Bowen’s disease, 0 % for KA and 0.8 % (18.6 per 1000 PY) for AK. The incidence analyses were then repeated among those with no history or pre-existing non-melanoma malignant skin lesions (*N* = 2,496). Of those, the cumulative incidence (and incidence rate) for all metastatic melanoma patients was 0 % for cuSCC and KA, 1.0 % (22.8 per 1000 PY) for BCC, 0.04 % (0.9 per 1000 PY) for Bowen’s disease, and 0.5 % (12.3 per 1000 PY) for AK. Of metastatic melanoma patients who had history or pre-existing skin lesion, the cumulative incidence (and incidence rate), during the first 6 months post diagnosis, was 0.6 % (15.5 per 1000 PY) for cuSCC, 8.3 % (212.3 per 1000 PY) for BCC, 0 % for Bowen’s disease and KA, and 2.9 % (70.7 per 1000 PY) for AK. Compared with those with no history or pre-existing skin lesion, the incidence of cuSCC, BCC and AK were higher, although these results were based on the small number (*N* = 314) of metastatic melanoma patients who had history or pre-existing skin lesion. We observed virtually no differences in cumulative incidence or incidence rate of non-melanoma malignant skin lesions between patients who were initially diagnosed with metastatic melanoma and those who were initially diagnosed with early stage melanoma and then progressed to stage IV at a later date.

**Table 4:** Cumulative incidence and incidence rate of non-melanoma malignant skin lesions among patients with metastatic melanoma^a^

Outcome	Event, n	Cumulative Incidence^b^ (95 % CI)	Incidence Rate^b^ (95 % CI)
*Among all patients (N = 2810* ^*c*^ *, regardless of whether they had a history or pre-existing skin lesion)*			
CuSCC			
Within 30 d after the index date	0	--	--
Within 60 d after the index date	1	0.04 (0.004-0.2)	2.3 (0.3-16.2)
Within 90 d after the index date	2	0.1 (0.02-0.2)	3.1 (0.8-12.5)
Within 180 d after the index date	2	0.1 (0.02-0.2)	1.7 (0.4-6.7)
Within 270 d after the index date	4	0.1 (0.05-0.3)	2.4 (0.9-6.4)
Within 365 d after the index date	7	0.2 (0.1-0.5)	3.3 (1.6-7.0)
Ever post index date	26	0.9 (0.6-1.3)	3.7 (2.5-5.4)
BCC			
Within 30 d after the index date	15	0.5 (0.3-0.9)	66.7 (40.2-110.6)
Within 60 d after the index date	26	0.9 (0.6-1.3)	59.6 (40.5-87.5)
Within 90 d after the index date	30	1.1 (0.7-1.5)	47.1 (33.0-67.4)
Within 180 d after the index date	50	1.8 (1.3-2.3)	42.5 (32.2-56.1)
Within 270 d after the index date	59	2.1 (1.6 - 2.7)	36.0 (27.9- 46.4)
Within 365 d after the index date	68	2.4 (1.9 - 3.0)	32.9 (25.9- 41.7)
Ever post index date	140	5.0 (4.2 - 5.8)	21.0 (17.8- 24.8)
Bowen’s disease			
Within 30 d after the index date	0	--	--
Within 60 d after the index date	0	--	--
Within 90 d after the index date	0	--	--
Within 180 d after the index date	1	0.04 (0.004-0.2)	0.8 (0.1-6.0)
Within 270 d after the index date	3	0.1 (0.03-0.3)	1.8 (0.6-5.6)
Within 365 d after the index date	3	0.1 (0.03-0.3)	1.4 (0.5-4.4)
Ever post index date	9	0.3 (0.2-0.6)	1.3 (0.7-2.4)
KA			
Within 30 d after the index date	0	--	--
Within 60 d after the index date	0	--	--
Within 90 d after the index date	0	--	--
Within 180 d after the index date	0	--	--
Within 270 d after the index date	1	0.04 (0.004-0.2)	0.6 (0.1-4.3)
Within 365 d after the index date	3	0.1 (0.03-0.3)	1.4 (0.5-4.4)
Ever post index date	10	0.4 (0.2-0.6)	1.4 (0.8-2.6)
AK			
Within 30 d after the index date	7	0.2 (0.1-0.5)	31.1 (14.8-65.2)
Within 60 d after the index date	13	0.5 (0.3-0.8)	29.7 (17.2-51.1)
Within 90 d after the index date	17	0.6 (0.4-0.9)	26.6 (16.5-42.8)
Within 180 d after the index date	22	0.8 (0.5-1.2)	18.6 (12.2-28.2)
Within 270 d after the index date	33	1.2 (0.8-1.6)	20.0 (14.2-28.1)
Within 365 d after the index date	41	1.5 (1.1-2.0)	19.6 (14.4-26.6)
Ever post index date	91	3.2 (2.6-3.9)	13.3 (10.8-16.3)
*Among patients who had no history or pre-existing skin lesion (N = 2496)*			
cuSCC			
Within 30 d after the index date	0	--	**--**
Within 60 d after the index date	0	--	**--**
Within 90 d after the index date	0	--	**--**
Within 180 d after the index date	0	--	**--**
Within 270 d after the index date	1	0.04 (0.004-0.2)	0.7 (0.1-4.8)
Within 365 d after the index date	3	0.1 (0.03-0.3)	1.6 (0.5-4.9)
Ever post index date	17	0.7 (0.4-1.1)	2.7 (1.7-4.3)
BCC			
Within 30 d after the index date	10	0.4 (0.2-0.7)	49.9 (26.9-92.8)
Within 60 d after the index date	14	0.6 (0.3-0.9)	36.0 (21.3-60.7)
Within 90 d after the index date	15	0.6 (0.4-1.0)	26.4 (15.9-43.8)
Within 180 d after the index date	24	1.0 (0.6-1.4)	22.8 (15.3-34.0)
Within 270 d after the index date	27	1.1 (0.7-1.5)	18.3 (12.6-26.7)
Within 365 d after the index date	30	1.2 (0.8-1.7)	16.1 (11.3-23.1)
Ever post index date	77	3.1 (2.5-3.8)	12.5 (10.0-15.7)
Bowen’s disease			
Within 30 d after the index date	0	--	--
Within 60 d after the index date	0	--	--
Within 90 d after the index date	0	--	--
Within 180 d after the index date	1	0.04 (0.004-0.2)	0.9 (0.1-6.7)
Within 270 d after the index date	1	0.04 (0.004-0.2)	0.7 (0.01-4.8)
Within 365 d after the index date	1	0.04 (0.004-0.2)	0.5 (0.1-3.8)
Ever post index date	5	0.2 (0.1-0.4)	0.8 (0.3-1.9)
KA			
Within 30 d after the index date	0	--	--
Within 60 d after the index date	0	--	--
Within 90 d after the index date	0	--	--
Within 180 d after the index date	0	--	--
Within 270 d after the index date	0	--	--
Within 365 d after the index date	1	0.04 (0.004-0.2)	0.5 (0.1-3.8)
Ever post index date	6	0.2 (0.1-0.5)	0.9 (0.4-2.1)
AK			
Within 30 d after the index date	4	0.2 (0.1-0.4)	19.9 (7.5-53.2)
Within 60 d after the index date	8	0.3 (0.2-0.6)	20.5 (10.3-41.0)
Within 90 d after the index date	10	0.4 (0.2-0.7)	17.6 (9.5-32.6)
Within 180 d after the index date	13	0.5 (0.3-0.9)	12.3 (7.2- 21.2)
Within 270 d after the index date	19	0.8 (0.5-1.2)	12.9 (8.2-20.2)
Within 365 d after the index date	23	0.9 (0.6-1.4)	12.3 (8.2-18.5)
Ever post index date	53	2.1 (1.6-2.7)	8.5 (6.5-11.1)

^a^Abbreviation: BCC, basal cell carcinoma; cuSCC, cutaneous squamous cell carcinoma; AK, actinic keratosis; and KA, keratoacanthoma

^b^The incidence analysis was conducted among 2810 patients, because 4 patients died on the index date and were excluded

^c^Cumulative incidence, %; incidence rate, per 1000 person-years

Of 2,810 metastatic melanoma patients, 3 patients developed non-cuSCC post metastatic melanoma diagnosis. Two non-cuSCC events were identified in the heart and the bronchi, respectively, during 30–60 days post metastatic melanoma diagnosis. The third event was identified in the lung during 90–180 days post metastatic melanoma diagnosis. The cumulative incidence was 0.1 % at 6 months after the index date, corresponding to an incidence rate of 2.5 per 1000 PY (Table 5).

**Table 5:** Cumulative incidence and incidence rate of non-cuSCC among patients with metastatic melanoma^a^

Outcome	Event, n	Cumulative Incidence^b^ (95 % CI)	Incidence Rate^b^ (95 % CI)
Within 30 d after the index date	0	--	--
Within 60 d after the index date	2	0.1 (0.02-0.2)	4.6 (1.1-18.2)
Within 90 d after the index date	2	0.1 (0.02-0.2)	3.1 (0.8-12.5)
Within 180 d after the index date	3	0.1 (0.03-0.3)	2.5 (0.8-7.8)
Within 270 d after the index date	3	0.1 (0.03-0.3)	1.8 (0.6-5.6)
Within 365 d after the index date	3	0.1 (0.03-0.3)	1.4 (0.5-4.4)
Ever post index date	3	0.1 (0.03-0.3)	0.4 (0.1-1.3)

^a^Abbreviation: non-cuSCC, non-cutaneous squamous cell carcinoma. The incidence analysis was conducted among 2810 patients, because 4 patients died on the index date and were excluded

^b^Cumulative incidence, %; incidence rate, per 1000 person-years

We further evaluated the cumulative incidence and incidence rates of BCC and AK among metastatic melanoma patients by age at metastatic melanoma diagnosis (Table 6). Incidence rates of BCC and AK were the lowest among the youngest patients between 18–65 years at metastatic melanoma diagnosis. The incidence rates of BCC (but not AK) increased with age at metastatic diagnosis, even among those aged 65 years and above. The age-stratified analyses were not possible for cuSCC, Bowen’s disease, KA, and non-cuSCC because the numbers of these events were too small.

**Table 6:** Incidence rate of BCC and AK by age at metastatic melanoma diagnosis^a^

Outcome	18– < 65 y		65– < 75 y		75- < 85 y		85+ y	
	N^b^	Rate (95 % CI)	N	Rate (95 % CI)	N	Rate (95 % CI)	N	Rate (95 % CI)
*Among all patients (n = 2810* ^*c*^ *, regardless of whether they had a history or pre-existing skin lesion)*								
BCC								
Within 30 d after the index date	7	59.4 (28.3-124.5)	2	39.5 (9.9-158.1)	2	46.8 (11.7-187.1)	4	291.2 (109.3-775.9)
Within 60 d after the index date	11	47.9 (26.5-86.5)	3	30.5 (9.8-94.6)	8	97.3 (48.6-194.5)	4	151.9 (57.0-404.8)
Within 90 d after the index date	11	32.8 (18.1-59.3)	6	41.8 (18.8-93.1)	8	66.8 (33.4-133.5)	5	131.8 (54.8-316.6)
Within 180 d after the index date	17	27.2 (16.9-43.8)	13	49.1 (28.5-84.5)	12	55.0 (31.2-96.8)	8	118.1 (59.0-236.1)
Within 270 d after the index date	19	21.7 (13.8-34.0)	16	43.4 (26.6-70.9)	16	52.6 (32.2-85.9)	8	87.3 (43.7-174.6)
Within 365 d after the index date	21	18.9 (12.4-29.1)	20	43.3 (27.9-67.1)	19	49.4 (31.5-77.4)	8	71.0 (35.5- 142.0)
Ever post index date	52	13.2 (10.0-17.3)	36	26.4 (19.1-36.6)	36	32.3 (23.3-44.8)	16	69.8 (42.7-113. 9)
AK								
Within 30 d after the index date	2	16.9 (4.2-67.6)	3	59.4 (19.1-184.0)	1	23.4 (3.3-165.9)	1	72.5 (10.2-515.0)
Within 60 d after the index date	2	8.7 (2.2-34.7)	5	51.0 (21.2-122.4)	4	48.4 (18.2-129.0)	2	75.3 (18.8-301.3)
Within 90 d after the index date	3	8.9 (2.9-27.6)	6	41.9 (18.8-93.2)	6	49.8 (22.4-110.9)	2	52.1 (13.0-208.3)
Within 180 d after the index date	3	4.8 (1.5-14.8)	10	37.7 (20.3-70.1)	6	27.3 (12.3-60.7)	3	43.3 (14.0-134.4)
Within 270 d after the index date	4	4.5 (1.7-12.1)	13	35.2 (20.4-60.5)	11	35.8 (19.8-64.7)	5	53.5 (22.3-128.5)
Within 365 d after the index date	6	5.4 (2.4-11.9)	16	34.4 (21.1-56.1)	14	35.9 (21.3-60.6)	5	43.5 (18.1-104.5)
Ever post index date	27	6.7 (4.6-9.7)	33	23.6 (16.8-33.3)	22	19.2 (12.6-29.2)	9	36.3 (18.9-69.7)
*Among patients, who had no history or pre-existing skin lesion (n = 2496)*								
BCC								
Within 30 d after the index date	4	36.0 (13.5-96.0)	2	45.5 (11.4-181.8)	2	56.9 (14.2-227.5)	2	198.0 (49.5-791.8)
Within 60 d after the index date	6	27.7 (12.5-61.7)	2	23.4 (5.8-93.4)	4	58.8 (22.1-156.8)	2	102.9 (25.7-411.6)
Within 90 d after the index date	6	19.0 (8.5-42.3)	3	24.0 (7.7-74.4)	4	40.2 (15.1-107.2)	2	71.4 (17.9-285.6)
Within 180 d after the index date	9	15.3 (8.0-29.4)	7	30.2 (14.4-63.3)	6	32.8 (14.8-73.1)	2	39.8 (10.0- 159.3)
Within 270 d after the index date	9	10.9 (5.7-21.0)	9	27.8 (14.5-53.5)	7	27.4 (13.1-57.5)	2	29.4 (7.3-117.4)
Within 365 d after the index date	11	10.5 (5.8-19.0)	9	22.1 (11.5-42.5)	8	24.6 (12.3-49.3)	2	23.8 (6.0-95.1)
Ever post index date	36	9.6 (6.9-13.3)	19	15.5 (9.9-24.3)	17	17.4 (10.8-28.0)	5	28.0 (11.7-67.4)
AK								
Within 30 d after the index date	2	18.0 (4.5-71.9)	2	45.5 (11.4-181.9)	0	--	0	--
Within 60 d after the index date	2	9.2 (2.3-36.9)	3	35.1 (11.3-108.8)	3	44.0 (14.2-136.4)	0	
Within 90 d after the index date	2	6.3 (1.6-25.3)	3	24.0 (7.7-74.4)	5	50.2 (20.9-120.7)	0	--
Within 180 d after the index date	2	3.4 (0.8-13.6)	5	21.6 (9.0-51.9)	5	27.4 (11.4-65.7)	1	19.6 (2.8-139.4)
Within 270 d after the index date	3	3.6 (1.2- 11.2)	7	21.6 (10.3-45.4)	8	31.3 (15.6-62.6)	1	14.5 (2.0-103.1)
Within 365 d after the index date	5	4.8 (2.0-11.4)	8	19.6 (9.8-39.2)	9	27.7 (14.4-53.2)	1	11.8 (1.7- 83.7)
Ever post index date	18	4.7 (3.0-7.4)	21	17.0 (11.1-26.1)	11	11.9 (6.1-20.0)	3	16.2 (5.2-50.3)

^a^Abbreviation: BCC, basal cell carcinoma; AK, actinic keratosis. Incidence rate, per 1000 person-years

^b^Number of events

^c^The analysis was conducted among 2810 patients, because 4 patients died on the index date and were excluded

## Discussion

This study provides population-based background rates of non-melanoma malignant skin lesions and non-cuSCC among over 2,800 metastatic melanoma patients in Denmark. The Danish national registries are unique resources for studying non-melanoma malignant skin lesions, as these are not generally captured in most cancer registries elsewhere.

The uniformly organized Danish healthcare system allows a truly population-based design. The DCR is population-based with mandatory notification of all incident cancers by hospitals, general practitioners and practicing specialists, and has a high degree of completeness. NPR, additionally, captures a large number of metastatic melanomas identified during the follow-up post initial diagnosis. Utilizing both national databases in Denmark allowed us to include not only metastatic melanoma patients with initial diagnoses of metastatic disease, but also those who had earlier stage melanoma initially then progressed to metastatic cutaneous melanoma. Compared with most randomized clinical trials, the results obtained from this study are relatively robust, given this sample size, and the metastatic melanoma patients included in this study should be fairly representative of those patients in the clinics, as only minor inclusion and exclusion criteria were applied.

Furthermore, the data quality of DCR and NPR is high [21–23]. Although the definition of diagnostic criteria or sensitivity of the diagnostic tools may have changed over time, which may explain the decrease in the proportion of patients with initial metastatic melanoma diagnosis over time, the validity measured by the proportion of morphologically verified tumors in DCR has been found to be 89 % [21], a very high proportion internationally [27]. Results from our validation of metastatic melanoma diagnosis in the NPR presented a nearly perfect positive predictive value. Therefore, we expect selection bias to be minimal. With the ability to link the DCR and the NPR to the CRS, which contains daily updated vital status of the entire Danish population, we had virtually no loss of follow-up for these patients. These national health registries in Denmark have been widely used in epidemiological studies [28]. Standard data quality checks are routinely performed and missing data and loss to follow-up are relatively minor issues for these national health registries.

This study has limitations. The completeness of the NPR is not known. Lesions that were not histological verified and were not reported to the cancer registry may have been missed, and therefore the incidence rates of non-melanoma skin lesions may be underestimated. Nonetheless, the NPR in Denmark contains all histological examinations, including those from office-based treatment facilities. Thus, our research captured most lesions with histological confirmation. An article published in 2010 by Birch-Johansen F et al. [29] showed the BCC and SCC incidence rates for Denmark in the period 1978–2007, using data from both DCR and NPR. The incidence rates reported in this paper were among the highest incidence rates ever found in a European country. The authors acknowledged that the two registries are not 100 % complete in capturing BCC/SCC, but suggest that the high rates may partially be due to a more complete registration of SCC and especially BCC in these registries compared to registries in other European countries.

We had limited statistical precision for several rare study outcomes, especially cuSCC, Bowen’s disease, KA, and non-cuSCC. This must be taken into consideration when interpreting the estimates. The results from this study may not be directly generalized to a different country, region, or ethnic group.

## Conclusion

In conclusion, our results suggest that metastatic melanoma patients may further develop non-melanoma skin lesions and non-cuSCC; however, the incidence is low. Among the tumor types studied in this Danish cohort, BCC was more common, followed by AK, cuSCC, and non-cuSCC. Bowen’s disease and KA were the most rare. These background rates provide population-based context for contextualizing safety signals from the randomized clinical trials. Furthermore, these results may inform physicians and patients when similar events are observed in the clinics among metastatic melanoma patients.

## Appendix

**Table 7:** ICD-10 Diagnosis Codes and SNOMED Morphology Codes for Non-melanoma Malignant Skin Lesions and Non-cutaneous Squamous-cell Carcinomas

	SNOMED morphology codes	ICD-10 codes
Metastatic melanoma	M87206, M87209, M87216, M87219, M87306, M87406, M87409, M87416, M87436, M87456, M874A6, M874A9, M87606, M87609	DC43
SCC	M80513, M80523, M80703, M80713 M80743 M80753 M80763	NA^a^
BCC	M80903, M80913, M80923, M80933, M80943, M80953	NA
Bowen’s disease	M80812	NA
KA	M72860	NA
AK	M72850	NA
Non-cuSCC	M80513, M80523, M80703, M80713 M80743 M80753 M80763	NA

^a^NA, not available or not relevant

## Abbreviations

AE: adverse event
AJCC: American Joint Committee on Cancer
AK: actinic keratosis
CRS: the Danish Civil Registration System
BCC: basal-cell carcinoma
cuSCC: cutaneous squamous-cell carcinomas
DCR: the Danish Cancer Registry
ICD: International classification of diseases
KA: keratoacanthoma
MAPK: mitogen-activated protein kinase
Non-cuSCC: non-cutaneous squamous-cell carcinomas
NPR: the National Pathology Registry
OS: overall survival
PFS: progression-free survival
PPV: positive predictive value
SAS: Statistical Analysis Software
